# Peripheral Nerve Injury-Induced Astrocyte Activation in Spinal Ventral Horn Contributes to Nerve Regeneration

**DOI:** 10.1155/2018/8561704

**Published:** 2018-04-03

**Authors:** Changhui Qian, Dandan Tan, Xianghai Wang, Lixia Li, Jinkun Wen, Mengjie Pan, Yuanyuan Li, Wutian Wu, Jiasong Guo

**Affiliations:** ^1^Guangdong Provincial Key Laboratory of Construction and Detection in Tissue Engineering, Southern Medical University, Guangzhou 510515, China; ^2^Department of Histology and Embryology, Southern Medical University, Guangzhou 510515, China; ^3^Department of Histology and Embryology, Fujian University of Traditional Chinese Medicine, Fuzhou 350000, China; ^4^Department of Anatomy, Li Ka Shing Faculty of Medicine, The University of Hong Kong, Pokfulam, Hong Kong; ^5^State Key Laboratory of Brain and Cognitive Sciences, The University of Hong Kong, Pokfulam, Hong Kong; ^6^Joint Laboratory of Jinan University and The University of Hong Kong, GHM Institute of CNS Regeneration, Jinan University, Guangzhou 510632, China; ^7^Institute of Bone Biology, Academy of Orthopedics, Guangzhou, Guangdong Province 510665, China

## Abstract

Accumulating evidences suggest that peripheral nerve injury (PNI) may initiate astrocytic responses in the central nervous system (CNS). However, the response of astrocytes in the spinal ventral horn and its potential role in nerve regeneration after PNI remain unclear. Herein, we firstly illustrated that astrocytes in the spinal ventral horn were dramatically activated in the early stage following sciatic nerve injury, and these profiles were eliminated in the chronic stage. Additionally, we found that the expression of neurotrophins, including brain-derived neurotrophic factor (BDNF), nerve growth factor (NGF), and neurotrophin-3 (NT-3), also accompanied with astrocyte activation. In comparison with the irreversible transected subjects, astrocyte activation and the neurotrophic upregulation in the early stage were more drastic in case the transected nerve was rebridged immediately after injury. Furthermore, administering fluorocitrate to inhibit astrocyte activation resulted in decreased neurotrophin expression in the spinal ventral horn and delayed axonal regeneration in the nerve as well as motor function recovery. Overall, the present study indicates that peripheral nerve injury can initiate astrocyte activation accompanied with neurotrophin upregulation in the spinal ventral horn. The above responses mainly occur in the early stage of PNI and may contribute to nerve regeneration and motor function recovery.

## 1. Introduction

Increasing evidences reveal that peripheral nerve injury (PNI) initiates many cellular responses in the central nervous system (CNS), including astrocyte activation [1–3]. Activated astrocytes in the spinal dorsal horn have been identified as one of the predominant factors that contributes to the development and maintenance of neuropathic pain following PNI [4–6]. However, existing literatures just roughly mentioned astrocytes in the spinal ventral horn would be activated by PNI [1, 7], while no detailed study on this issue is reported, and the potential roles of activated astrocytes in peripheral nerve regeneration remain very poorly defined. It is known that the spinal dorsal horn and ventral horn have quite different morphological and functional profiles. The dorsal horn contains interneurons and receives the sensory terminals from the peripheral nerve, while the ventral horn contains motoneurons whose axons are extending into the peripheral nerve. So the pattern and roles of PNI-initiated astrocyte activation in the spinal ventral horn should be different to those in the dorsal horn. The motoneurons in the ventral horn must be attacked in the acute stage of PNI and then will gradually recovery. Astrocytes are predominant glial cells for surrounding and supporting neurons [8, 9]. Astrocytes and neurons are always keeping cross talk with each other no matter in physiological or pathological states [10]. Therefore, we speculate astrocytes in the spinal ventral horn would respond to the axotomized motoneurons, and the response might be different in acute and chronic stages; moreover, the response of astrocytes might play a role in the motoneurons' capability of regeneration.

In order to verify our hypothesis and to collect data for understanding the potential role of astrocytes in axonal regeneration following PNI, we designed the present study. Herein, we first illustrated the temporal astrocyte activation in the spinal ventral horn at different stages following PNI as well as the neurotrophic expression in the activated astrocytes. Then, differences between irreversible PNI model and regenerable PNI model were analyzed. Finally, the potential role of activated astrocytes in axonal regeneration was assessed after an inhibitor (fluorocitrate) was administered to attenuate astrocyte activation.

## 2. Materials and Methods

### 2.1. Animals

Adult female Sprague-Dawley (SD) rats (180–200 g) were used for this project. All rats were housed under a 12 h light/dark cycle and given free access to water and food. The procedures of this study were performed in accordance with NIH guidelines for the care and use of laboratory animals and approved by the Animal Experimental Ethics Committee of Southern Medical University, Guangdong Province, China. All efforts were made to minimize animal suffering and usage.

### 2.2. Location of the Axotomized Motoneurons in the Spinal Ventral Horn by Fluoro-Gold (FG) Retrograde Labeling

To illustrate the range of axotomized motoneurons in the spinal cord that were involved in the sciatic nerve injury, 4 rats were used to perform FG retrograde labeling. Briefly, the sciatic nerves of each rat were exposed and transected at the mid-thigh level under intraperitoneal anesthesia with 2,2,2-tribromoethanol (90 mg/kg). And then, tiny gelatin sponge soaked with 3 *μ*l of 5% FG solution (Sigma) was tied to the proximal end of the transected nerve. Seven days later, the spinal cord (L5 segment) was harvested after perfusion fixation and cut into samples with a 20 *μ*m thickness in a cryostat (Leica).

### 2.3. Preparation of the Irreversible PNI Model and Regenerable PNI Model

After intraperitoneal anesthesia, the sciatic nerves of rats were exposed and transected at the mid-thigh level. The right nerve was immediately sutured in an end-to-end manner to work as regenerable PNI model, while a 5 mm nerve segment in the sciatic nerve was removed to form an irreversible PNI model [11]. Following the surgery, the rats were survived for 7, 14, 28, or 56 days (*n* = 9, for each time point). For sham control, only the nerve was exposed, but no PNI was performed.

### 2.4. Fluorocitrate Administration to Inhibit Astrocyte Activation in the Spinal Cord

To inhibit astrocytic reactions in the spinal cord to assess the roles of activated astrocytes in axonal regeneration, fluorocitrate (a widely used astrocytic-specific inhibitor) [12–14] was administered intrathecally according to Jasmin and Ohara's procedure with modifications [15]. In brief, an intrathecal catheter (PE-10 tube) was inserted into the subarachnoid space at the L4-L5 level. Three days later, 10 *μ*l of 2% lidocaine was intrathecally injected through the catheter; only the animals that showed paralysis for approximately 30 min and then recovered without neurologic deficits were selected as qualified animals for further analysis. The day after the lidocaine test, the sciatic nerve at the front-thigh level was crushed with a microforceps for 2 min. Then, 10 *μ*l fluorocitrate (1 nM, Sigma) (*n* = 15) or saline (*n* = 15) was administered daily via the reserved catheter for 3 consecutive days. Four hours after the last drug administration, 9 animals of each group were euthanized for detecting axonal regenerative rate by western blotting (*n* = 3 for each group) and immunohistochemistry (*n* = 6 for each group). Ten days after the last drug administration, 6 animals of each group were subjected to perform behavior test to assess the motor function recovery.

### 2.5. Behavior Testing

At the designed time points, footprint assessment was carried out to evaluate the functional recovery of the injured nerves. Briefly, subjected animals were trained to walk along a glass gallery (120 cm length, 7 cm width, and 8 cm height). Foot contact points of each rat were captured by a video recorder (Mouse Specifics Inc., DigiGait), and the sciatic functional index (SFI) was calculated and analyzed from the captured images according to previous protocol [16–18].

### 2.6. Tissue Preparation

At the designed time points, 6 rats from each group were euthanized, and the segment of the spinal cord at L5 as well as the sciatic nerves were collected after routine perfusion. The tissues were postfixed in 4% paraformaldehyde (PFA) for 24 h, dehydrated in 30% sucrose for 48 h at 4°C, and then cryosectioned with a thickness of 10 *μ*m. All sections were continuously mounted onto poly-l-lysine-treated slides and archived at −20°C for further immunohistochemistry. Meanwhile, the gastrocnemius muscles were dissected and weighed.

### 2.7. Immunohistochemistry

Every one-tenth of the continuous sections of each sample was used to perform immunohistochemistry. Briefly, after being washed with PBS, the sections were penetrated by 1% Triton X-100 for 1 h and incubated with blocking buffer at room temperature (RT) for 1 h. Subsequently, the tissues were incubated with primary antibodies overnight at 4°C, fluorescent 488- or 568-conjugated secondary antibodies (1 : 400) for 2 h at RT, and 4′,6-diamidino-2-phenylindole (DAPI, 1 : 2000, Sigma, D9542) for 2 min. Finally, the sections were coverslipped with Vectashield (Vector). The following primary antibodies were used: mouse anti-glial fibrillary acidic protein (GFAP, 1 : 500, Abcam, ab10062), rabbit anti-neuron-specific nuclear protein (NeuN, 1 : 400, Abcam, ab177487), rabbit anti-brain-derived neurotrophic factor (BDNF, 1 : 400, Abcam, ab6201), rabbit anti-nerve growth factor (NGF, 1 : 200, Millipore, 04-1119) or rabbit anti-neurotrophin-3 (NT-3, 1 : 200, Santa Cruz, SC-547) for spinal cord sections, and rabbit anti-growth-associated protein 43 (GAP-43, 1 : 500, Abcam, ab16053) for sciatic nerve sections.

### 2.8. Morphometric Analysis

**Figure 1:**
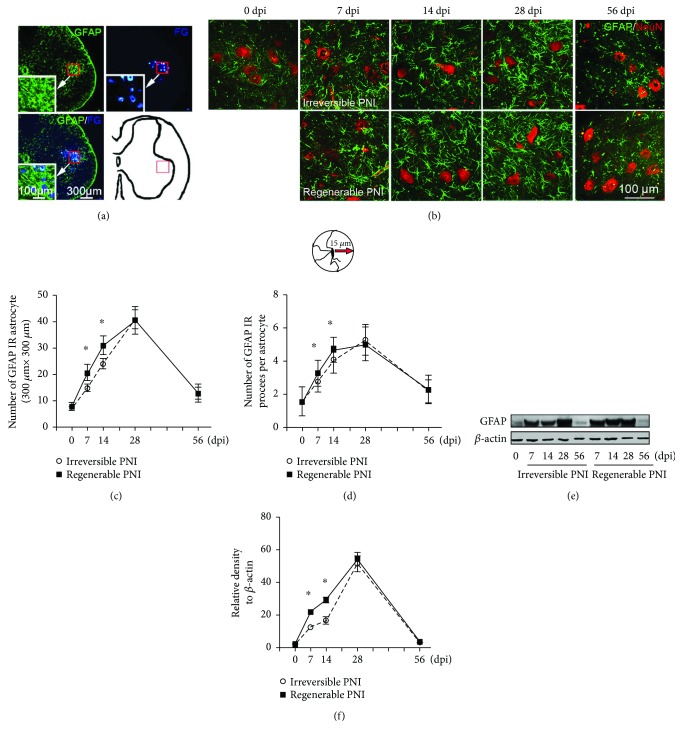
The pattern of astrocyte activation in the spinal ventral horn after sciatic nerve injury. (a) Representative cross sections of L5 spinal cord showing GFAP-positive astrocytes and FG retrograde-labeled motoneurons that are mainly localized in the spinal ventral horn. So, a schematic diagram indicating a 300 *μ*m × 300 *μ*m square area was defined for further morphometric analysis. (b) GFAP/NeuN double immunostaining showing the activated astrocytes (green) and motoneurons (red) in the area as (a) defined in different groups. (c) Quantitative analysis of the number of GFAP-positive astrocytes in each measured area. (d) The number of GFAP-positive processes (measured at 15 *μ*m away from the soma) in each astrocyte. (e-f) Immunoblot analysis of GFAP expression in the L5 spinal ventral horn. ^∗^*P* < 0.05.

GFAP and neurotrophin expressions were analyzed on a 300 *μ*m × 300 *μ*m square area (Figure 1(a)), which was defined by the abovementioned FG labeling experiment as most sciatic nerve corresponding motoneurons are located in. After, the images of immunostained spinal cord were captured under a fluorescence microscope (Leica, DMI 4000B), the number of GFAP-positive astrocytes in each restricted square was counted and the number of processes of each astrocyte was quantified to indicate the size of the individual astrocyte based on the method of Tyzack et al. [19], the immunointensities of neurotrophins in each astrocyte were assessed according to Sung et al.'s protocol [20], and the longest regenerating axon of the sciatic nerve after fluorocitrate inhibition was analyzed as reported previously [21, 22].

### 2.9. Western Blotting

Three rats of each group were decapitated at each time points; the L5 spinal cord was dissected and frozen at −80°C for 30 seconds, then the ventral horn tissues were separated carefully under a stereomicroscope based on the color and localization. The collected tissue was minced and homogenized in RIPA lysis buffer (Sigma) containing 1% protease inhibitor cocktail (Cell Signaling). Proteins were separated on 10% sodium dodecyl sulfate polyacrylamide gels and then transferred to a polyvinylidene difluoride membrane (Bio-Rad). After being blocked with 5% bovine serum albumin in Tris-buffered solution containing 0.5% Tween-20 for 2 h, blots were probed overnight at 4°C with the primary antibody of GFAP, followed by HRP-conjugated secondary antibodies for 2 h at room temperature. Immunoreactive proteins were visualized by electrochemiluminescence reaction and the band density was calculated using Image-Pro Plus 6.0 software.

### 2.10. Statistical Analysis

SPSS 20.0 software (IBM, USA) was used to conduct all statistical analyses. All values were expressed as the mean ± standard deviation. Two-way repeated-measures ANOVA followed by Bonferroni's multiple comparison test was used to analyze the difference of each time point between regenerable PNI and irreversible PNI groups. Student's *t*-test was used to compare the data between saline and fluorocitrate-treated groups. A value of *P* < 0.05 was considered statistically significant.

## 3. Results

### 3.1. Astrocytes in the Spinal Ventral Horn Are Activated by Peripheral Nerve Injury in Early Stages and Reverted in Later Stages

**Figure 2:**
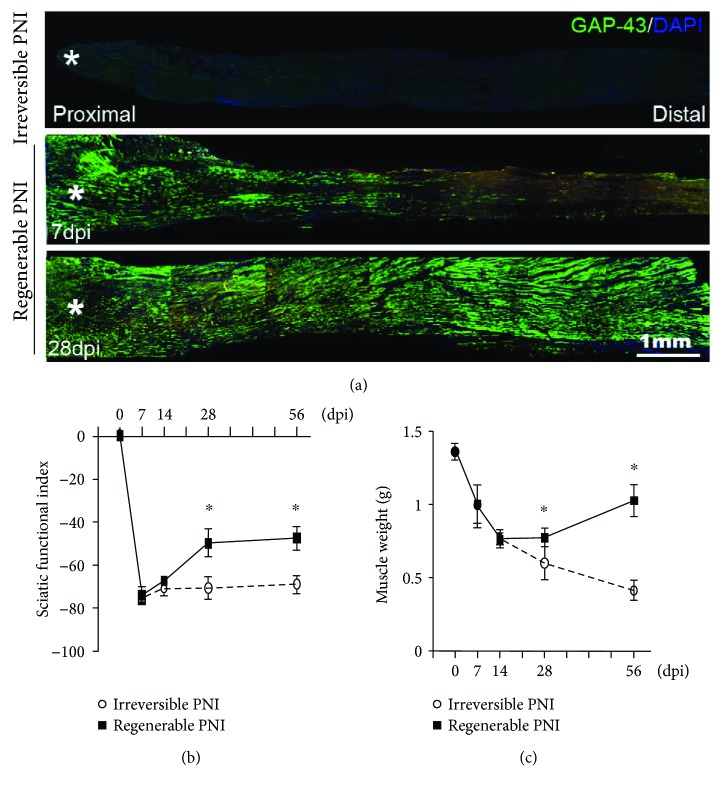
Identification of the PNI models. (a) Representative images of GAP-43 immunostained longitudinal sections to show no regenerating axon in the distal trunk of transected sciatic nerve at 28 dpi and the regenerating axons in the sutured nerves at 7 dpi and 28 dpi (^∗^transected sites). (b) Statistics of the sciatic functional index and (c) weight of gastrocnemius muscle assessments in the ipsilateral side of the injured nerve. ^∗^*P* < 0.05.

In this study, the transected nerve could represent an irreversible PNI injury as no any axonal regeneration and functional recovery could be detected (Figure 2). On the other hand, the sutured nerve represented regenerable PNI model as morphological and functional recovery was detected at 14 days postinjury (dpi) and achieved a relatively high level at 28 dpi and 56 dpi (Figure 2).

Then, FG retrograde labeling was applied to show the motoneurons in the spinal ventral horn. Incorporating the GFAP immunostaining, a widely accepted specific marker of activated astrocyte [23], we found the activated astrocytes surrounded the FG-labeled motoneurons (Figure 1(a)). In order to standardize the following quantifications for analyzing astrocyte activation, a 300 *μ*m × 300 *μ*m square area (Figure 1(a)) was defined within the range of motoneurons and activated astrocytes in the spinal ventral horn, and the morphometric analyses were performed in this restricted area.

As shown in Figures 1(b)–1(d), few GFAP-positive astrocytes could be detected in the sham animal, while both astrocyte density and the number of processes per astrocyte were dramatically increased following PNI. These increasing patterns lasted from 7 dpi to 28 dpi and then reverted on 56 dpi. Moreover, at 7 dpi and 14 dpi, astrocyte density and the number of processes were significantly higher in the sutured group as compared to the transected group. Not surprisingly, the change of GFAP protein level in the spinal ventral horn revealed by western blotting also appeared similar patterns as above (Figures 1(e) and 1(f)).

### 3.2. Neurotrophins Overexpressed in the Activated Astrocytes

**Figure 3:**
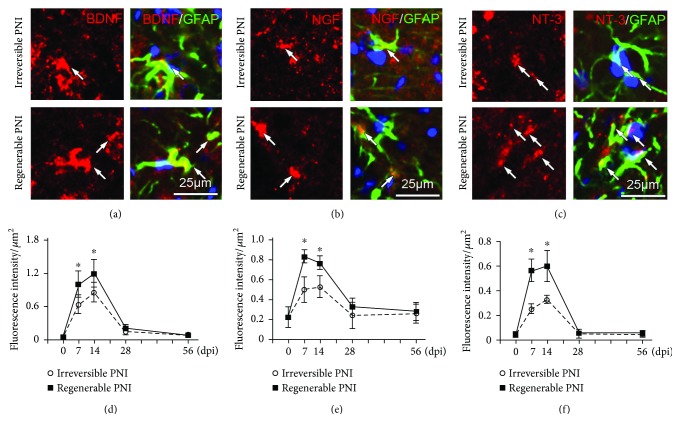
Immunohistochemistry showing the expression of neurotrophins in the spinal ventral horn. (a–c) Representative images of immunostaining of BDNF, NGF, and NT3 on the samples of regenerable PNI group at 14 dpi to show the expression of neurotrophins (arrows) within astrocytes. (d–f) Statistical diagrams showing the fluorescence intensities of neurotrophins within the GFAP-positive astrocytes at different time points. ^∗^*P* < 0.05.

To assay the expression level of neurotrophins in the spinal ventral horn, double immunostaining with antibodies of neurotrophins and GFAP was performed and the results revealed that neurotrophin immunoreactivities were found to predominantly colocalize with GFAP-positive astrocytes (including soma and processes) (Figures 3(a)–3(c)). The histomorphometric analysis showed that the neurotrophin immune intensity within astrocytes was significantly increased at 7 dpi and 14 dpi. Moreover, the neurotrophin levels of the sutured group were higher than that of the transected group at 7 and 14 dpi (Figures 3(d) and 3(e)).

### 3.3. Inhibiting Astrocyte Activation in the Spinal Cord Retarded Axonal Regeneration and Motor Function Recovery

**Figure 4:**
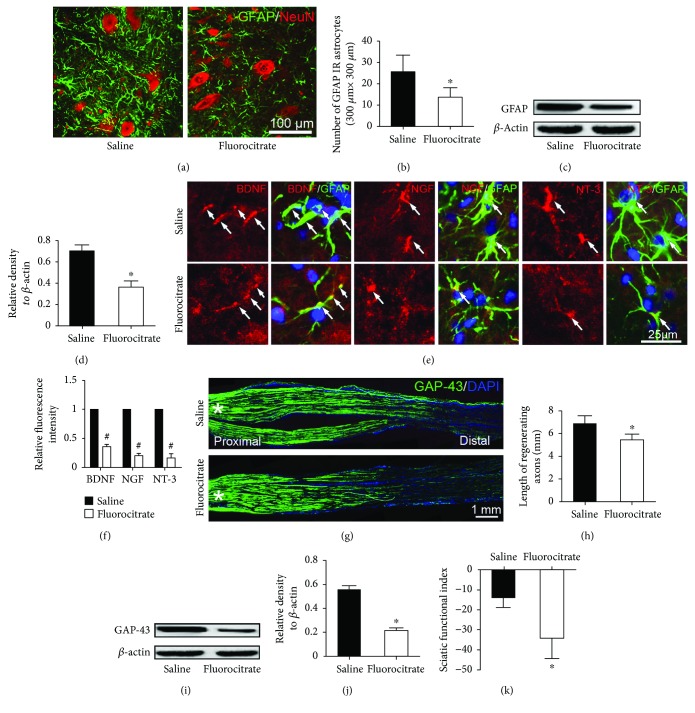
Fluorocitrate treatment inhibits astrocyte activation and reduces neurotrophin expression in the spinal ventral horn and retards nerve regeneration as well as motor function recovery. (a-b) Immunohistochemistry and quantification illustrating that the number of the activated astrocytes was significantly decreased in the fluorocitrate-treated group. (c-d) Western blots revealing that the level of GFAP in the spinal ventral horn was also markedly reduced in the fluorocitrate-treated group. (e) Immunohistochemistry showing the neurotrophins (arrows) colocalizing with GFAP-positive in the spinal ventral horn. (f) Statistical diagram showing that the intensity of neurotrophin immunoreactivity in the fluorocitrate-treated group was significantly reduced. (g) GAP-43 immunochemistry showing the regenerated axons in the injured nerve (^∗^lesion site). (h) Statistics showing the length of regenerated axons. (i–j) Western blots and quantification showing the GAP-43 expression level in the distal segment of the injured nerve. (k) Statistics of the sciatic functional index assessments in the injured nerve. ^∗^*P* < 0.05 versus saline group, ^#^*P* < 0.05 versus the standardized value of saline group.

In order to explore the potential role of the activated astrocytes in peripheral nerve regeneration, a widely used astrocyte inhibitor (fluorocitrate) was administered to inactivate the astrocyte response. Following fluorocitrate administration, the inhibition of astrocyte activation was verified by immunohistochemistry and western blotting. As displayed in Figures 4(a)–4(d), both the density of GFAP-positive astrocytes and the GFAP protein level in the spinal ventral horn were dramatically decreased in the fluorocitrate-treated group as compared to the saline control group. Meanwhile, immunohistochemistry also showed that the expression levels of neurotrophins were significantly reduced by fluorocitrate treatment (Figures 4(e) and 4(f)). Then, the GAP-43 antibody was used to identify the regenerating axons at 3 dpi. In the immunostained sciatic nerve, the length of GAP-43-positive axons was significantly shorter in the fluorocitrate-treated group as compared with the saline control group (Figures 4(g) and 4(h)). Western blotting of the distal segment of the injured sciatic nerve also showed that the protein level of GAP-43 in the fluorocitrate-treated group was decreased (Figures 4(i)–4(j)). Moreover, motor functional behavior test demonstrated the SFI of fluorocitrate-treated groups is statistically lower than that of control group (Figure 4(k)).

## 4. Discussion

Most of the peripheral nerves, especially spinal nerves, are mixed nerves which contain motor nerve fibers and sensory nerve fibers. The axons of sensory nerve fibers are derived from the sensory neurons in the dorsal root ganglions while the motor axons are from the motoneurons in the spinal ventral horn. As for PNI, motor functional recovery is one of most important aspects for the patients, while the recovery depends on axonal regeneration from the motoneurons [24, 25]. Even the peripheral nerve has considerable capacity for regeneration compared to CNS, but the functional repair is far from the expected level which mainly due to the axonal regenerative rate is quite slow [26]. The existing studies mostly target to neurons and/or environment of lesion site [27, 28]; no evidence reveals the relationship of peripheral nerve regeneration and astrocytes in the CNS up to date. To our knowledge, the present report is the first study to illustrate the temporal response of astrocytes in the spinal ventral horn and its potential role in axonal regeneration after PNI. Herein, we firstly demonstrated that astrocytes in the spinal ventral horn were dramatically activated in the early stage following sciatic nerve injury, and these profiles are eliminated in the chronic stage. Moreover, we found that the expression of neurotrophins, including BDNF, NGF and NT-3, also accompanied with astrocyte activation. In case of nerve rebridge immediately after transection, the axons in the injured nerve were still having an ongoing regeneration at 7 dpi and 14 dpi as shown in immunostaining with GAP-43 antibody (Figure 2(a)), and no functional outcomes as shown in the behavior assessment (Figure 2(b)) and target muscle test (Figure 2(c)). At 28 dpi and 56 dpi, the regenerated axons fulfilled distal nerve trump, and the motor function and weight of target muscles showed significant recovery compared to the irreversible nerve injury (a segment of nerve was cut off from the injury site); these data indicate the regenerated axons had reached and reinnervated target muscles after 28 dpi. Overall, the present data hint astrocyte activation, and neurotrophin upregulation in the spinal ventral horn occurs mainly in the nerve regenerative stage, and then astrocytes will gradually return to their normal state after the functional nerve regeneration was achieved.

To further explore the potential relationship between astrocytic activation with nerve regeneration, we compared the regenerable PNI (the transected nerve was rebridged) with irreversible PNI (the nerve was defected with a 5 mm gap). Statistical data showed that astrocyte activation and the neurotrophic upregulation in the early stage were more drastic in the regenerable PNI group, which implies the regenerating motoneurons require more activated astrocytes compared with merely axotomized motoneurons.

As astrocyte activation highly positively correlates with nerve regeneration and activated astrocytes highly express neurotrophins, we assumed that the activated astrocytes might play a pivotal role in the neighboring motoneurons to facilitate its axonal regeneration via paracrine neurotrophins. As expected, administering fluorocitrate to inhibit astrocyte activation resulted in decreased neurotrophin expression in the spinal ventral horn and delayed axonal regeneration in the nerve lesion as well as the motor function recovery.

Overall, the present study demonstrates that (i) peripheral nerve injury can initiate astrocyte activation in the spinal ventral horn, but the activation mainly occurs in the early stage of PNI and will evanish in the chronic stage; (ii) the neurotrophin expression is upregulated in the activated astrocytes; (iii) the astrocyte activation and neurotrophin upregulation are more significant in case the injured nerve is having an ongoing regeneration than irreversible injury; and (iv) the inhibition of astrocyte activation retards nerve regeneration in the nerve lesion as well as weakens the neurotrophin expression. Based on the above data, we conclude that PNI can initiate astrocyte activation accompanied with neurotrophin upregulation in the spinal ventral horn. The above responses mainly occur in the early stage of PNI and may contribute to nerve regeneration and motor function recovery.

## Abbreviations

BDNF: Brain-derived neurotrophic factor
CNS: Central nervous system
DAPI: 4′,6-diamidino-2-phenylindole
dpi: Days postinjury
FG: Fluoro-Gold
GAP-43: Growth-associated protein 43
GFAP: Glial fibrillary acidic protein
NeuN: Neuron-specific nuclear protein
NGF: Nerve growth factor
NT-3: Neurotrophin-3
PBS: Phosphate-buffered saline
PFA: Paraformaldehyde
PNI: Peripheral nerve injury
RT: Room temperature
SD: Sprague-Dawley
SFI: Sciatic functional index.
